# VASERP: An Adaptive, Lightweight, Secure, and Efficient RFID-Based Authentication Scheme for IoV

**DOI:** 10.3390/s23115198

**Published:** 2023-05-30

**Authors:** Yinyan Gong, Kuanching Li, Lijun Xiao, Jiahong Cai, Jiahong Xiao, Wei Liang, Muhammad Khurram Khan

**Affiliations:** 1 School of Computer Science and Engineering, Hunan University of Science and Technology, Xiangtan 411201, China; 2 Hunan Key Laboratory for Service Computing and Novel Software Technology, Xiangtan 411201, China; 3 College of Information Engineering, Shanghai Maritime University, Shanghai 201306, China; 4 Center of Excellence in Information Assurance, King Saud University, Riyadh 11653, Saudi Arabia

**Keywords:** authentication, ECC, RFID, Scyther, IoV

## Abstract

With the rapid growth in wireless communication and IoT technologies, Radio Frequency Identification (RFID) is applied to the Internet of Vehicles (IoV) to ensure the security of private data and the accuracy of identification and tracking. However, in traffic congestion scenarios, frequent mutual authentication increases the overall computing and communication overhead of the network. For this reason, in this work, we propose a lightweight RFID security fast authentication protocol for traffic congestion scenarios, designing an ownership transfer protocol to transfer access rights to vehicle tags in non-congestion scenarios. The edge server is used for authentication, and the elliptic curve cryptography (ECC) algorithm and the hash function are combined to ensure the security of vehicles’ private data. The Scyther tool is used for the formal analysis of the proposed scheme, and this analysis shows that the proposed scheme can resist typical attacks in mobile communication of the IoV. Experimental results show that, compared to other RFID authentication protocols, the calculation and communication overheads of the tags proposed in this work are reduced by 66.35% in congested scenarios and 66.67% in non-congested scenarios, while the lowest are reduced by 32.71% and 50%, respectively. The results of this study demonstrate a significant reduction in the computational and communication overhead of tags while ensuring security.

## 1. Introduction

RFID [1] is one of the fundamental technologies in the Internet of Things (IoT) [2]. It can identify specific targets and read relevant data through radio signals without the need for direct contact with the targets. The Internet of Vehicles is an important component of the Internet of Things, in which vehicles can exchange data with roadside infrastructure. Such a network can help drivers better grasp traffic information and ensure road safety [3,4]. The rapid development of IoT [5,6] has driven the application of RFID in IoV. RFID has been used in various IoV scenarios, such as vehicle location tracking and traffic prediction [7,8,9,10]. Any roadside unit (RSU) must meet contactless and rapid identification requirements and read information from passing vehicles. RFID tags can be embedded in vehicles and associated with other vehicle information, such as vehicle identity and location information, to identify and track vehicle location and movement.

During vehicle identification, location and driving information can be leaked or falsified [11,12,13], and RFID is vulnerable to attacks during wireless transmission. To ensure the accuracy of IoV traffic information, RFID authentication is required before using the reader for identification reading. This is crucial to ensure that the privacy data from the vehicle network remain secure and that information identification remains accurate. RFID authentication for IoV has become an important issue for many scholars. Based on different identification methods, RFID tags can be divided into active and passive tags, as well as semiactive tags. Active and passive tags have very limited storage capacity and computing power [14,15], which poses a greater security threat to RFID tags. Therefore, RFID authentication protocols must be lightweight and secure to provide effective protection.

**Figure 1:**
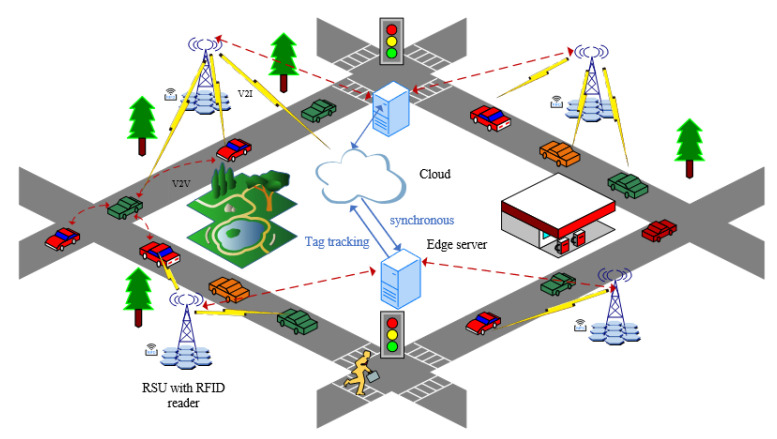
RFID authentication for IoV framework.

When traffic is congested, vehicles tend to stay in one place for long periods of time. As a result, the RSU and the vehicle will perform frequent mutual authentication, which can lead to a significant amount of computation and communication overhead. However, longer authentication intervals can reduce security, so it is important to balance security and overhead by reducing the authentication time. In non-congested scenarios, vehicles move faster, and RFID authentication needs to be performed quickly to ensure that the reader on the RSU can read all vehicle tags within the range [16]. Figure 1 illustrates the RFID authentication framework for IoV. The contributions of this paper are as follows.

- This paper presents an edge server-based RFID authentication protocol for IoV, which reduces the computing and communication costs of the cloud due to the high overhead of cloud computing, communication, and storage;
- The protocol proposed in this research is the first to achieve fast authentication in the IoV traffic congestion scenario, significantly lower computing and communication overhead, and to satisfy security and lightweight authentication requirements.

## 2. Related Work

RFID can be utilized in the IoV to enable vehicle identification and authentication functions, providing technical support for smart transportation and smart city construction. While RFID allows for automatic identification and management without contact, its non-contact feature also poses certain security risks. Therefore, the RFID authentication protocol plays a critical role in ensuring the secure transmission of vehicle network data and accurate identification information. Many scholars have contributed to research on RFID authentication, which mainly includes lightweight authentication protocols based on bit operations, RFID authentication protocols based on quadratic residue, ECC, and grouping-proof, and other RFID authentication protocols.

(1) Lightweight RFID authentication protocols based on bit operations
  To reduce computational and communication overhead in the RFID authentication process, several scholars have proposed lightweight RFID protocols based on bit operations. Chen et al. [17] introduced a lightweight authentication protocol based on an asymmetric encryption algorithm, where the reader performs most of the complex work, and the tag only needs to execute simple operations such as bitwise XOR, one-bit circular shift, and bit flip. This method reduces computational and communication overhead. Fan et al. [18] proposed a lightweight RFID authentication privacy protection scheme that encrypts authentication data through the operation of a cross and the operation of rotation. Although the computational overhead of this algorithm is relatively small, it is vulnerable to attacks. Aghili et al. [19] introduced a lightweight authentication protocol and designed a more secure function to encrypt private data in the authentication process by improving the function. Fan et al. [20] proposed an efficient protocol that uses rotation and enhanced permutation encryption to reduce RFID overhead. However, the ambiguous timestamp in this protocol is vulnerable to brute-force attacks, leading to secret information leakage. Although these protocols are lightweight, they use simple bit operations such as XOR, bit flip, and rotation to encrypt private data, which can be vulnerable to attacks and may result in key leakage.
(2) RFID authentication protocols based on quadratic residue
  Scholars have implemented RFID mutual authentication using the quadratic residue algorithm to improve security. Fan et al. [21] proposed a lightweight authentication protocol based on the quadratic residue and the operation of rotation. However, the computational speed of the quadratic residue is slow, leading to high computational overhead, which does not meet the lightweight requirements. Doss et al. [22] proposed a grouping-proof authentication protocol based on the quadratic residue zero-knowledge property. However, in this protocol, the reader is not authenticated, which can make it vulnerable to counterfeit readers that illegally read the tag data. Lee et al. [23] proposed a protocol based on homomorphic encryption and quadratic residue to address the issue of ownership transfer in the RFID of the IoV, which can achieve batch ownership transfer of tags. However, Tu et al. [24] conducted a security assessment of the protocol and identified security loopholes that could result in attacks such as replay, tracking, and simulated tag attack. To address these issues, Song et al. [25] proposed a quadratic residue-based zero-knowledge authentication protocol with active tags that have zero-knowledge properties.
(3) RFID authentication protocols based on grouping-proof
  To achieve fast authentication in large-scale RFID systems, Rahman et al. [26] proposed a group-based anonymous privacy authentication protocol to achieve batch authentication. This method is better than the original tree-based authentication protocol, and the group-based protocol security level is higher. To address the security challenge of simultaneous authentication of multiple readers and tags in a distributed RFID system, Liu et al. [27] proposed a Grouping-Proofs-Based Authentication Protocol (GUPA) that can withstand classic attacks such as replay, forgery, and tracking. However, Sun et al. [28] pointed out that the GUPA scheme [27] is vulnerable to man-in-the-middle attacks and can result in key leakage, leading to replay, forgery, tracking, and rejection of proofs. Therefore, Sun et al. [28] utilized a hash function to encrypt private data for better authentication security. To solve the problem of high communication overhead when authenticating tags in batches, Yang et al. [29] proposed an efficient authentication grouping-proof protocol based on symmetric keys and bit collisions, which can solve the conflict issue of one-time authentication of multiple tags.
(4) RFID authentication protocol based on ECC
  Wu et al. [30] introduced a UAV-assisted IoV authentication protocol that utilizes ECC and hash functions for encryption. Analysis shows that the protocol can withstand various attacks, but its computational and communication overheads are relatively high. Shabani et al. [31] proposed an ECC-based RFID authentication protocol for IoV, but this protocol may result in tag tracking. Kumar et al. [32] proposed an ECC-based RFID authentication protocol for vehicle cloud computing, RSEAP, which is resistant to man-in-the-middle attacks and replay attacks and has high-performance communication. However, Safkhani et al. [33] pointed out that the protocol proposed by Kumar et al. could not provide the claimed security or improved vulnerability based on RSEAP. They therefore proposed a more secure RSEAP2 protocol with less computing and communication overhead, but with more overhead in authentication. During the process, neither the tag nor the cloud will verify the identity of the reader, which may lead to man-in-the-middle attacks and impersonation of the reader attacks. Meher et al. [34] proposed an ECC-based RFID authentication protocol without public/private key pairs to address the issue of limited resources of RFID tags, greatly reducing the computation and storage costs of the tags.
(5) Other RFID authentication protocols
  Chander et al. [35] proposed an RFID protocol based on hash functions and bit logic functions, which are easy to implement but vulnerable to attacks. Jangirala et al. [36] proposed a blockchain RFID protocol based on a single hash function, bit-by-bit dissimilarity, and bit-by-bit rotation operations, enhancing security and increasing communication overhead. Salem et al. [37] proposed a privacy-preserving RFID authentication protocol based on ElGamal encryption. The protocol allows for direct authentication between the tag and server without the need for the reader’s involvement. Unlike traditional authentication methods in which both the tag and the server authenticate each other, in this protocol, only the tag authenticates the server. However, it also makes the protocol sensitive to beacon attacks simulated by potential attackers. Shariq et al. [38] proposed a Schnorr-based RFID authentication protocol. However, the protocol provides only one-way verification of the server and does not verify the legitimacy of the tag. Akram et al. [39] proposed an RFID authentication protocol based on cloud computing for IoV. The protocol utilizes a chaotic map to achieve mutual authentication and offers significant advantages in terms of computing, communication, and storage compared to other protocols. However, the mutual authentication process within the protocol is cloud-based, which may overload computing, communication, and cloud storage.

Current RFID authentication schemes typically rely on cloud-based authentication, resulting in high computing and communication overheads. This can lead to reduced real-time performance, decreased network reliability, and an increased risk of data leakage. To address these challenges, this paper proposes an RFID authentication for IoV that utilizes edge servers, thereby reducing the cloud burden. Although many high-security encryption algorithms cannot be applied directly to RFID due to the limited computing and storage capabilities of RFID tags, some encryption algorithms can still be used, such as hash functions and ECC algorithms. Hash functions have the advantages of fast calculation speed and high security, making them a suitable option for RFID; however, using only a hash function can leave RFID vulnerable to attacks such as man-in-the-middle attacks. On the other hand, the ECC algorithm offers fast calculation speed and high security, making it a suitable choice for resource-constrained RFID systems [40,41]. ECC can protect private data in the authentication process and also offers a faster calculation speed.

To address the challenges of a heavy cloud computing communication burden, limited RFID resources, and the balance between RFID security and performance, this work proposes a lightweight and adaptive RFID security authentication protocol for IoV. The Elliptic Curve Discrete Logarithm Problem (ECDLP) of the ECC algorithm and the anti-collision characteristics of the hash function are utilized to guarantee the security of the protocol. This scheme balances security and performance by ensuring low overhead calculation, communication, and storage in the authentication process.

The remainder of this paper is organized as follows. The preparation work for this study is introduced in Section 3, covering the ECC algorithm and the adversary model. In Section 4, an IoV-based, lightweight, adaptive, and efficient RFID security authentication scheme is proposed, while the security of this proposed scheme is analyzed in Section 5. Performance comparison and evaluation are demonstrated in Section 6. Finally, this work is summarized, and the directions for future work are discussed in Section 7.

## 3. Preliminaries

### 3.1. ECC Algorithm over Finite Fields

The ECC algorithm over finite fields [42] is an asymmetric encryption algorithm. Let GF(p) be a finite field on a large prime number *p*. The most commonly used curve equation for elliptic curves over finite fields is defined below.

(1) $$y^{2}\equiv x^{3}+ax+b\left(\;\mathrm{mod}\;p\right)\left(a,b,x,y\in GF\left(p\right),4a^{3}+27b^{2}\neq 0\right)$$

Let $E_{p}$ be an elliptic curve over a finite field GF(p) of large prime numbers *p*. Define $G_{+}=\left\{\left(x,y\right):x,y\in GF\left(p\right);\left(x,y\right)\in E_{p}\cup O\right\}$, which is an additive group, and *O* is an element in $E_{p}$ called the point at infinity, which is also the identity element of $G_{+}$. Let $P,Q\in E_{p}$, have the following definition:

1. P+O=P;
2. Let P=(x,y), then P+(−P)=O, where −P=(x,−y) represents the additive inverse of *P*;
3. Let Q=kP, then $Q=\underset{\underset{k}{⏟}}{P+P+...+P}$;
4. Let $P=\left(x_{1},y_{1}\right),Q=\left(x_{2},y_{2}\right)$, P≠−Q, then $P+Q=(x_{3},y_{3})$, $x_{3},y_{3}$ is determined by:
  (2) $$x_{3}\equiv \lambda^{2}-x_{1}-x_{2}\left(\;\mathrm{mod}\;p\right)$$
  (3) $$y_{3}\equiv \lambda \left(x_{1}-x_{3}\right)-y_{1}\left(\;\mathrm{mod}\;p\right)$$
  where
  (4) $$\lambda =\left\{\begin{array}{c} \frac{y_{2}-y_{1}}{x_{2}-x_{1}},P\neq Q\\ \frac{3x_{1}+a}{2y_{1}},P=Q \end{array}\right.$$

### 3.2. Difficult Problems

**Definition** **1** (ECDLP). *Given two points P and Q on an elliptic curve $E_{p}$, computing a positive integer k such that Q=kP is difficult in polynomial time.*

**Definition** **2** (Elliptic Curve Diffie–Hellman Problem (ECDLP)). *Given two points A and B on an elliptic curve $E_{p}$, and knowing A=aP and B=bP($P\in E_{p}$), solving C=abP in polynomial time is difficult.*

**Definition** **3** (One-way Property of the Hash Function). *Given M=h(m), it is difficult to solve m.*

**Definition** **4** (Collision Resistance of Hash Functions). *Given different plaintexts $m_{1},m_{2}$, the probability of $h\left(m_{1}\right)=h\left(m_{2}\right)$ is negligible; given $M=h(m_{1})$, finding $m_{2}$ such that $M=h(m_{2})$ is difficult.*

### 3.3. Adversary Model

To analyze the security of the authentication scheme proposed in this paper, certain assumptions must be made regarding the adversary’s attack capabilities. This section introduces attack models commonly used in many related studies [27,28,29], which will be used for security analysis. The assumptions of the adversary model are as follows:

(1) An adversary can interrupt communication between readers, tags, and edge servers through common channels;
(2) The adversary in this paper can launch active and passive attacks against the protocol;
(3) Adversaries can launch attacks using fake readers or tags.

## 4. The VASERP Protocol

The paper presents a scheme involving four entities: the cloud server, the edge server, the vehicle, and the RSU. The tag, which contains information such as vehicle identity, location, and status, is embedded in the vehicle, while the RFID reader is installed in the RSU to identify and read information from passing vehicles. Both tags and readers register through the cloud, and the cloud records their identifiers. The cloud does not directly synchronize the tag identifiers with the edge server to ensure data privacy. Instead, it encrypts the hash and then synchronizes it with the edge server. The edge server performs the verification between RFIDs. In a special situation that requires verification of the vehicle’s real identity, the edge server can send a request to the cloud server.

During RFID authentication, the reader will process the information on the tag to give road condition information and forecast traffic conditions to prevent illegal tags from forcing vehicles to provide false road condition information. During authentication, the tag needs to be authenticated, and in order to ensure that the information about the vehicle is not read by an illegal reader, the reader also needs to be authenticated. At the same time, in order to ensure that the read information is non-repetitive, this paper stipulates that, at a certain moment, one reader can read multiple tags at the same time, but one tag can be read by only one reader. Three stages are included: (1) the registration phase, (2) the fast mutual authentication phase, and (3) the ownership transfer phase. Algorithm 1 is the pseudocode for an adaptive selection protocol scheme that can choose appropriate authentication protocols based on the actual traffic scenarios
**Algorithm 1:** An adaptive, lightweight, secure, and efficient RFID-based authentication scheme for IoV.$Tag_{i},R_{j}$ Register with the cloud; the cloud synchronizes the RID to the edge server after hashing and encryption$R_{j}$ broadcast $M_{1}:\left\{Query,R_{j1},R_{j2},T_{1}\right\}$ to the surrounding area, where$R_{j1}=n_{r}G$,$R_{j2}=RID_{j}⊕\;n_{r}P$.$Tag_{i}$ receive $M_{1}:\left\{Query,R_{j1},R_{j2},T_{1}\right\}$if $T_{2}-T_{1}\leq \Delta T$   compute $RID{}_{j}=R_{j2}⊕kR_{j1}$   if $rid==RID_{J}$      Perform rapid mutual authentication phase   else      Perform the transfer of ownership phaseelse   return 0 (Request Timeout)

### 4.1. Initialization Phase

An appropriate elliptic curve should be chosen to implement the authentication protocol, and a large prime number *d* should be generated randomly as the private key of the edge server. The public key Q=dG can then be calculated through the base point *G* of the curve. Table 1 provides symbols and definitions used in the authentication protocol.

### 4.2. Registration Phase

This phase aims to establish the legitimate identities of the readers and tags in the system, with registration carried out through a secure channel. Upon registration, a unique identifier will be obtained for the reader or tag, which will be stored in the cloud to facilitate subsequent real identity tracking. To protect the privacy of vehicle information, the edge server will only store encrypted tag identifier information. As shown in Table 2, to verify the legal identity of the tag and record the authentication status of the tag, the edge server will maintain a tag index table.

#### 4.2.1. Tag Registration

Step1: $Tag_{i}$ generates its own identifier $TID_{i}$ and sets a variable rid to store the reader identifier of the tag currently accessible, initially rid=null, then sends a registration request {registration request,$TID_{i}$,$Tt_{i}$} to the cloud through a secret channel.

Step2: The cloud receives registration request, $TID_{i}$, $Tt_{i}$, and checks whether the same value as $TID_{i}$ exists already in Table 2. If it exists, it means that $Tag_{i}$ has already been registered, and the registration fails. Otherwise, it creates a record in Table 2. The cloud sends {*k*, *p*, *G*, *Q*} to $Tag_{i}$. The cloud encrypts the identifier of the tag through the hash and synchronizes it to each edge server.

#### 4.2.2. Reader Registration

Step1: $R_{j}$ sends a registration request to the cloud {registration request $R_{j}$}

Step2: The cloud receives registration request $R_{j}$ and then sends {$RID_{j}$, *k*, *G*, *Q*} to the reader.

### 4.3. Fast Mutual Authentication Phase

This phase is a mutual rapid verification process designed for the traffic congestion scene of the IoV. It achieves quick mutual authentication of tags and readers without the need for an edge server, thus reducing the computational and communication overhead associated with authentication in the congestion scenario of the IoV. Specific steps for rapid mutual authentication are outlined below.

Step1: $R_{j}$ generates a random number $n_{r}\in Z_{p}^{*}$; calculate:

(5) $$R_{j1}=n_{r}G$$

(6) $$R_{j2}=RID_{j}⊕n_{r}P$$

which broadcasts around $M_{1}:\left\{Query,R_{j1},R_{j2},T_{1}\right\}$.

Step2: Nearby tags receive $M_{1}:\left\{Query,R_{j1},R_{j2},T_{1}\right\}$ and verify $T_{2}-T_{1}\leq \Delta T$; if valid, then compute:

(7) $$RID{}_{j}=R_{j2}⊕kR_{j1}$$

Formula (7) indicates that only a legitimate tag with a shared key *k* can solve $RID_{j}$ based on $M_{1}$. Then, it checks $rid==RID_{J}$. If it is true, it means that $R_{j}$ and $T_{i}$ have been authenticated by the edge server, and the fast authentication between the reader and the tag can be performed directly. The tag computes:

(8) $$T_{i1}=h\left(RID_{j}⊕k\right)⊕n_{r}P$$

Send $M_{2}:\left\{T_{i1},T_{3}\right\}$ to the reader. If false, then perform the ownership transfer phase.

Step3: Reader $R_{j}$ receives $M_{2}:\left\{T_{i1},T_{3}\right\}$ and verifies $T_{4}-T_{3}\leq \Delta T$; if valid, then compute:

(9) $$R_{j3}=kR_{j1}$$

(10) $$R_{j4}=h\left(RID_{j}⊕k\right)$$

Verify $R_{j4}\overset{?}{=}\;R_{j3}⊕T_{i1}$, while $\overset{?}{=}\;$ represents the operation ==. Since $RID_{j}$, *k*, and $n_{r}P$ are all secret information, they are not available to illegitimate tags and attackers. Only legitimate tags can obtain them using formula (7). Therefore, if it is true, the tag is a legal tag; otherwise, it is an illegal tag. Then calculate:

(11) $$R{}_{j5}=h\left(RID_{j}⊕k⊕n_{r}P\right)$$

and send $M_{5}:\left\{R_{j5},T_{5}\right\}$ to the validated tag.

Step4: $Tag_{i}$ receives $M_{5}:\left\{R_{j5},T_{5}\right\}$ and verifies $T_{6}-T_{5}\leq \Delta T$; if it is valid, then verify $R{}_{j5}\overset{?}{=}\;h\left(RID_{j}⊕k⊕n_{r}P\right)$, and if it is valid, then compute $n_{r}$ and verify the authenticity of the value of $n_{r}$ through the value of $n_{r}G$. If the verification passes, it is a legal reader, and fast mutual authentication ends.

**Figure 2:**
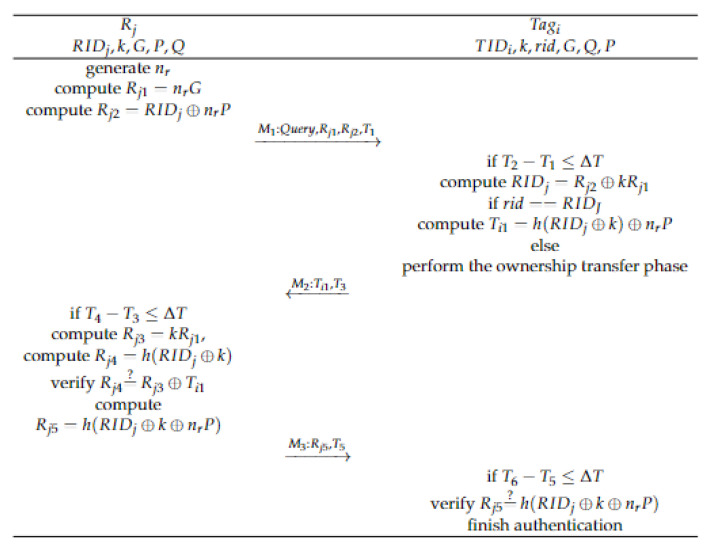
The fast mutual authentication phase.

The process of fast mutual authentication is shown in Figure 2.

### 4.4. The Ownership Transfer Phase

This phase is applied to non-congested scenarios in IoV. In this scenario, the vehicle tags are mutually authenticated with the readers in the RSU. When a vehicle moves from one reader’s reading range to another, an ownership transfer is required. This process relies on edge servers to transfer the ownership of the tag. That is, authentication between the reader and the tag is done through the edge server. The following are the detailed steps for ownership transfer:

Step1: $Tag_{i}$ generates a random number $n_{t}\in Z_{p}^{*}$; calculate:

(12) $$T_{i1}=n_{t}G$$

(13) $$T_{i2}=h(RID_{j}\left||k|\right|T_{i1})$$

(14) $$T_{i3}=h\left(TID_{i}\right)⊕n_{t}Q$$

and send $M2:\{T_{i1},T_{i2},T_{i3},T_{3}\}$ to $R_{j}$. Formula (13) is used by the reader to verify the identity information of the ${Tag}_{i}$, while formula (14) is used by the edge server to verify the identity information of ${Tag}_{i}$.

Step2: $R_{j}$ receives $M2:\{T_{i1},T_{i2}$,$T_{i3},T_{3}\}$ and verifies $T_{4}-T_{3}\leq \Delta T$. If it is legal, it verifies the validity of $Tag_{i}$ by verifying $T_{i2}\overset{?}{=}\;h(RID_{j}\left||k|\right|T_{i1})$. If it is legal, it calculates:

(15) $$R_{j3}=n_{r}Q$$

(16) $$R_{j4}=RID_{j}+R_{j3}$$

(17) $$R_{j5}=h(RID_{j}\left|\right|R_{j3}\left||k\right)$$

and sends $M_{3}:\left\{T_{i1},T_{i3},R_{j1},R_{j4},R_{j5},T_{5}\right\}$ to $S_{k}$.

Step3: $S_{k}$ receives $M_{3}:\left\{T_{i1},T_{i3},R_{j1},R_{j4},R_{j5},T_{5}\right\}$ and verifies $T_{6}-T_{5}\leq \Delta T$. If it is legal, it computes:

(18) $$E_{1}=dT_{i1}$$

(19) $$E_{2}=dR_{j1}$$

(20) $$h\left(TID_{i}\right)=E_{1}⊕T_{i3}$$

(21) $$RID_{j}=E{}_{2}⊕R_{j4}$$

$S_{k}$ has the private key *d* of the edge server, so it can obtain $RID_{j}$ through Formulas (19) and (21) and can verify $RID_{j}$ by checking if $R_{j5}\overset{?}{=}h(RID_{j}\left||E2||k\right)$. In addition, $S_{k}$ calculates $h(TID_{i})$ using Formulas (18) and (20). To verify $Tag_{i}$, $S_{k}$ searches $h(TID_{i})$ in Table 2. If the search fails, it means that the tag is illegal; otherwise, update Table 2 and update the content corresponding to the $h(TID_{i})$ index value to $h\left(TID_{i}\right)⊕RID_{j}$. Then, generate a random number $n_{e}$ and calculate:

(22) $$E_{3}=h(RID_{j}\left|\right|E_{2}\left|\right|n_{e})$$

(23) $$E_{4}=RID_{j}⊕E_{1}$$

and send $M_{4}:\;\;\;\left\{\;\;\;E_{3},E_{4},n_{e},T_{7}\;\;\;\right\}\;\;\;$ to $R_{j}$, where $E_{3}$ is used for $R_{j}$ to verify $S_{k}$ and $E_{4}$ is used for $Tag_{i}$ to verify $S_{k}$.

Step4: $R_{j}$ receives $M_{4}:\;\;\;\left\{\;\;\;E_{3},E_{4},n_{e},T_{7}\;\;\;\right\}\;\;\;$ and verifies $T_{8}-T_{7}\leq \Delta T$. Since $RID_{j}$ and $R_{j3}$ are secret messages and $S_{k}$ can only calculate $R_{j3}$ using its private key *d* through formula (19), attackers and illegal devices cannot calculate $R_{j3}$. Therefore, the legitimacy of $S_{k}$ can be verified by checking $E_{3}\overset{?}{=}h(RID_{j}\left|\right|R_{j3}\left|\right|n_{e})$. If it is legitimate, then compute:

(24) $$R_{j6}=h\left(kT_{i1}⊕RID_{j}⊕n_{e}\right)$$

$R_{j}$ sends $M_{5}:\;\;\;\left\{\;\;\;E_{4},R_{j6},n_{e},T_{9}\;\;\;\right\}\;\;\;$ to ${Tag}_{i}$, where $R_{j6}$ is used for $Tag_{i}$ to verify $R_{j}$.

Step5: $Tag_{i}$ receives $M_{5}:\;\;\;\left\{\;\;\;E_{4},R_{j6},n_{e},T_{9}\;\;\;\right\}\;\;\;$ and verifies $T_{10}-T_{9}\leq \Delta T$. If it is true, then verify $E_{4}\overset{?}{=}\;h\left(n_{e}⊕n_{t}Q\right)$ to verify the validity of $S_{k}$ and verify $R_{j6}\overset{?}{=}\;h\left(kT_{i1}⊕TID_{j}⊕n_{e}\right)$ to check the the validity of $R_{j}$. As $n_{t}Q$ is a secret message, attackers cannot obtain it through $T_{i3}$. Moreover, due to the difficulty of ECDHP, attackers cannot solve $n_{t}Q$ from $n_{t}G$ and Q=dG. Only $S_{k}$ with the private key *d* can solve $n_{t}Q$. Set $rid=RID_{j}$. The mutual authentication of $R_{j}$ and $T_{i}$ is complete.

**Figure 3:**
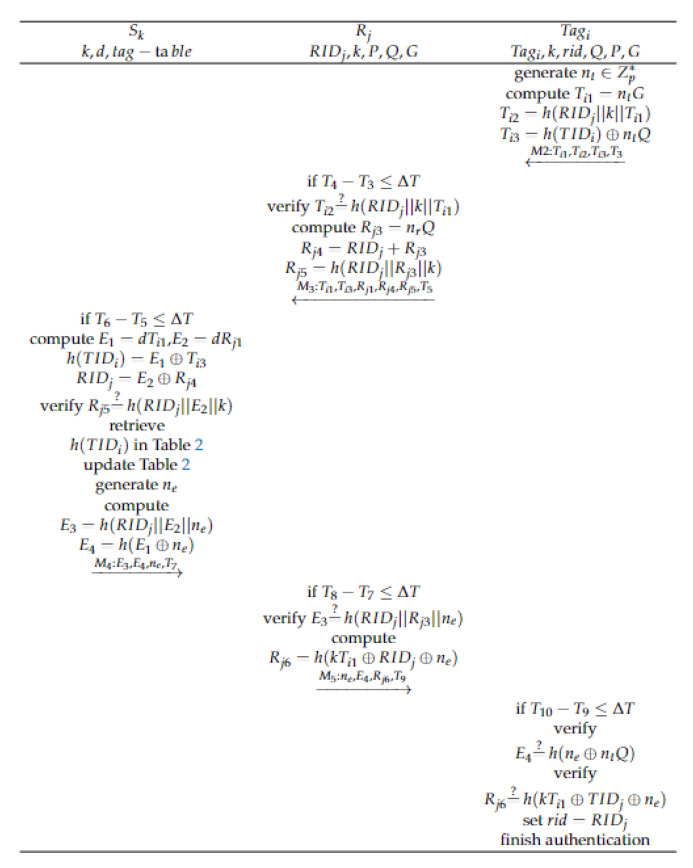
The ownership transfer phase.

The proposal of the ownership transfer phase is shown in Figure 3.

## 5. Security Analysis

### 5.1. Formal Analysis

This section presents a formal analysis of the protocol proposed in this paper to verify its security. Existing formal analysis methods include proof methods based on logical reasoning such as BAN logic and GNY logic, hypothesis-based formal proof methods such as random oracles, and automated formal proof methods such as Scyther and AVISPA. In this paper, we conduct a formal analysis of the proposed protocol using Scyther, an automated security protocol verification tool that can simulate attackers with varying levels of sophistication and detect potential vulnerabilities and flaws in security protocols. Scyther can also analyze security attributes such as confidentiality, integrity, authentication, and availability of the protocol and provide detailed analysis reports. The protocol must be written in a formal description language called SPDL for Scyther.

**Figure 4:**
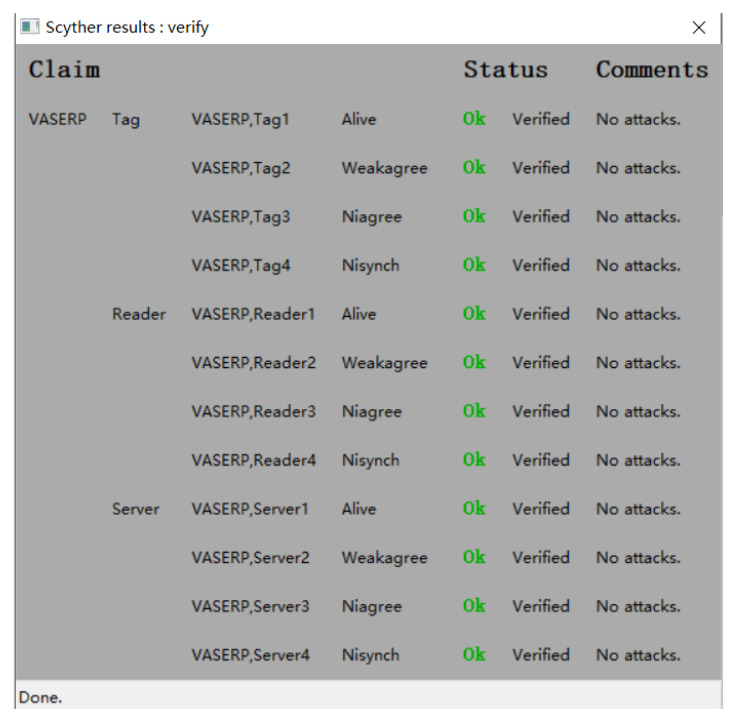
Scyther’s claim results for the VASERP.

The protocol defines participants using the roles of Tag, Reader, and Server. Each role can perform operations such as sending and receiving messages and computing key values. The claim section is used to verify the security properties of the protocol. Running the protocol code produces an analysis results window. When Scyther executes the proposed protocol, it begins by simulating adversaries with the Dolev–Yao model. If the protocol meets the verification requirements for security and no security vulnerabilities or attack types are found, Scyther outputs “OK”. If an attack is detected, Scyther outputs the type of attack, the role played by the attacker, and a flow chart of the attack. As shown in Figure 4, the protocol of this work satisfies security requirements and does not have vulnerabilities.

### 5.2. Informal Analysis

The following will provide an informal analysis of the scheme, mainly analyzing whether VASERP can withstand common attacks and whether it can meet the required security.

Support for tag anonymity: The tag is registered with the cloud through a secure channel, and the cloud will synchronize the $TID_{i}$ of $Tag_{i}$ with each edge server after hash encryption. When performing authentication, the edge server only knows $h(TID_{i})$ and cannot know the real identity of $Tag_{i}$. During the authentication process, the tag identifier will not appear directly in the message. Therefore, our scheme can support tag anonymity.

Achieve mutual authentication: Taking the ownership transfer stage as an example, $Tag_{i}$ sends $M2:T_{i1},T_{i2},T_{i3}$ to $R_{j}$, and $R_{j}$ verifies $Tag_{i}$ through $T_{i2}\overset{?}{=}\;h(RID_{j}\left||k|\right|T_{i1})$. $R_{j}$ sends $M_{3}:T_{i1},T_{i3},R_{j1},R_{j4},R_{j5}$ to $S_{k}$. $S_{k}$ obtains $h(TID_{i})$ though $T_{i1},T_{i3}$, then $S_{k}$ verifies whether $h(TID_{i})$ is in Table 2. $S_{k}$ obtains $RID_{j}$ by $R_{j1},R_{j4}$, and verifies $RID_{j}$ by checking $R_{j5}\overset{?}{=}\;h(RID_{j}\left|\right|E_{2}\left||k\right)$. $S_{k}$ sends $M_{4}:E_{3},E_{4},n_{e}$ to $R_{j}$, then $R_{j}$ verifies $E_{3}\overset{?}{=}\;h(RID_{j}\left|\right|R_{j3}\left|\right|n_{e})$. $R_{j}$ sends $M_{5}:E_{4},R_{j6},n_{e}$ to $Tag_{i}$, then $Tag_{i}$ verifies $S_{k}$ by checking $E_{4}\overset{?}{=}\;h\left(n_{e}⊕n_{t}Q\right)$ and verifies $R_{j}$ by checking $R_{j6}\overset{?}{=}\;h\left(kT_{i1}⊕TID_{j}⊕n_{e}\right)$. Therefore, the proposed scheme in this work can realize mutual authentication.

Provide key agreement: Take the ownership transfer stage as an example. $Tag_{i}$ and $R_{j}$ through $n_{r}P=kn_{r}G$ and $kn{}_{t}G=kn_{t}G$ perform mutual authentication. $Tag_{i}$ and $S_{k}$ conduct mutual authentication through $n_{t}Q=dn_{t}G$. $R_{j}$ and $S_{k}$ conduct mutual authentication through $n_{r}Q=dn_{r}G$. The dynamism of the session key depends on the random numbers $n_{t}$ and $n_{r}$. Due to the difficulty of ECDLP, it is difficult for an attacker to break the session key.

Even if *P*, *Q*, and *G* are leaked, the VASERP can prevent the imitation attack of tags: In the fast authentication phase, if an attacker wants to simulate a tag, they need to forge the information $M_{2}:T_{i1}$, where $T_{i1}=h\left(RID_{j}⊕k\right)⊕n_{r}P$. The attacker needs to obtain the secret messages $RID_{j}$, *k*, and $n_{r}P$. Since solving ECDLP is difficult, the attacker cannot solve $n_{r}P$ based on $R_{j1}$ in message $M_{2}$ (the fast authentication phase) and therefore cannot obtain $RID_{j}$. The key *k* is a shared secret between the reader and the tag that the attacker cannot obtain. Therefore, the attacker cannot simulate the tag. Similarly, the attacker cannot simulate the tag in the ownership transfer phase either.

VASERP is resistant to man-in-the-middle attacks: The authentication process of this scheme will verify the sender at each step, and the messages are encrypted by the hash function and ECC. If the attacker wants to steal the communication message in the authentication and manipulate it, then the attacker needs to obtain the secret information $k,d,TID_{i},RID_{j}$ and, at the same time, obtain the random numbers $n_{e},n_{r},n_{t}$ generated by the three authentication participants; however, these messages are not available to the attackers. Therefore, VASERP is resistant to man-in-the-middle attacks.

VASERP is resistant to replay attacks: In the fast authentication phase and the ownership transfer phase, the time stamp is set $T_{i}$, and the random number for each authentication round will be updated. Even if an attacker obtains a message in the communication, the attacker cannot find the session key, so the attacker cannot perform a replay attack using a previously eavesdropped message.

VASERP is resistant to desynchronization attacks: In the authentication process, in order to resist desynchronization attacks, this scheme sets a timestamp for each piece of information during authentication. After receiving the information, each entity will verify the timestamp to ensure the legitimacy of the message. If the timestamp expires, then the entity discards the message. An attacker cannot perform a desynchronization attack.

VASERP is resistant to tag tracking: In the authentication process, an attacker can eavesdrop on communication between the tag and the reader, and even if they do not know the real identity of the tag, they may be able to track it through repeated messages. In the fast authentication phase, the tag does not have identity information involved in the encrypted messages sent by the tag, and $T_{i1}$ changes with the value of $n_{r}$, which is a random number generated by the reader $R_{j}$ and which is different for each round of authentication. Therefore, $T_{i1}$ is also different for each round, and the attacker cannot track the tag according to $T_{i1}$. In the ownership transfer phase, the tag sends $M_{2}:T_{i1},T_{i2},T_{i3},T_{3}$ to the reader, where $T_{i1}=n_{t}G$, $T_{i2}=h(RID_{j}\left||k|\right|T_{i1})$, and $T_{i3}=h\left(TID_{i}\right)+n_{t}Q$. Only $T_{i3}$ contains the identity information of the tag $T_{i}$, but since $n_{t}$ is updated in each round of authentication, $T_{i3}$ is also updated, and the attacker cannot track the tag based on $T_{i3}$ unless the attacker can obtain $n_{t}Q=n_{t}dG$ through Q=dG and $T_{i1}=n_{t}G$. However, as defined in Definition 1, ECDLP is difficult. Therefore, the attacker cannot track the tag.

## 6. Performance Comparison and Discussion

In order to evaluate the performance of the proposed scheme, this paper compares the vehicular RFID protocol with the schemes proposed by Shabani et al. [31], Kumar et al. [32], Safkhani et al. [33], and Salem et al. [34] in terms of computation, communication, security, and storage in two scenarios: traffic congestion and non-congestion in vehicular networks.

### 6.1. Security Comparison

In order to better evaluate the security of the protocol, the protocol proposed in this work and other RFID protocols for IoV are evaluated from multiple security aspects, such as tag anonymity and resistance to replay attacks. It can be seen in Table 3 that, among the four protocols compared, only the proposed protocol and the scheme [33] can meet the security requirements stated in this investigation. Neither the protocols of reference [31] nor reference [32] can satisfy replay attacks, man-in-the-middle attacks, or impersonation attacks.

### 6.2. Computational Overhead Comparison

**Figure 5:**
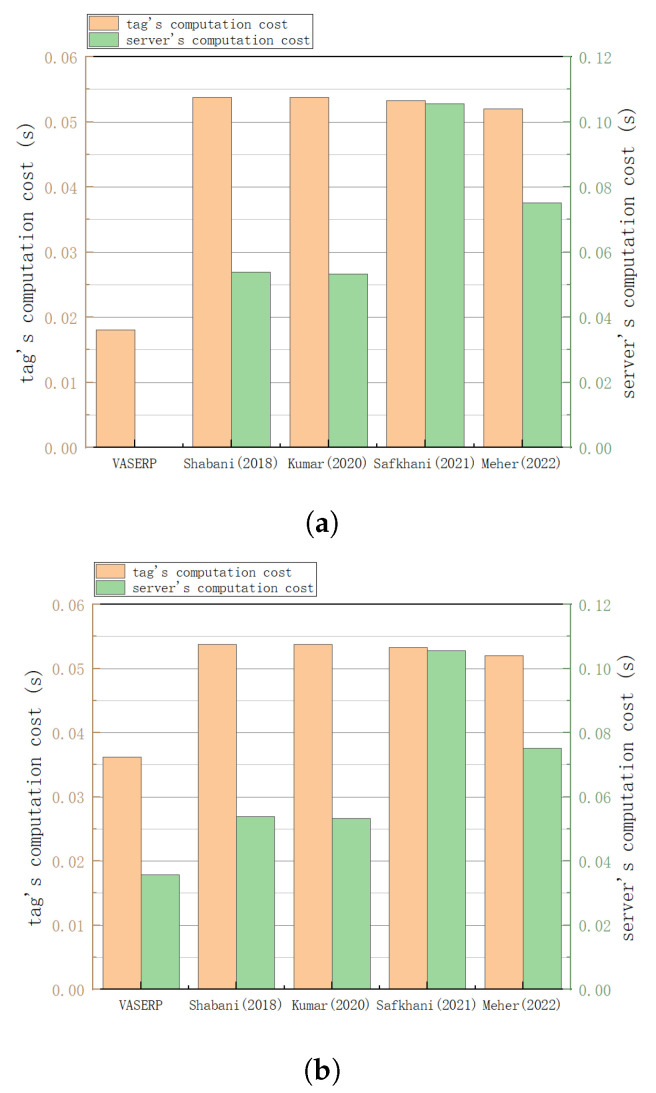
Computational overhead comparison. (**a**) Comparison of computational costs in congested scenarios; (**b**) comparison of computational costs in non-congested scenarios [31,32,33,34].

The computing capability of RFID tags is very limited, and computation and communication overhead is an important performance evaluation standard for RFID authentication protocols. This section compares different RFID protocols. Let $T_{h}$ be the time for SHA hash operations, $T_{ECM}$ be the time for elliptic curve point multiplication, $T_{ECA}$ be the time for elliptic curve addition, and $T_{sym}$ be the time for symmetric encryption/decryption. Amin et al. [40,43] used MIRACL, a C/C++ library, to estimate the computation time of different encryption methods. The authors in [40,43] used Visual C++ 2008 S/W and a 32-bit Windows 7 operating system to calculate the encryption time of 1024 cyclic groups, 160-bit prime fields, 160-bit elliptic curve addition, and the SHA-1 hash function. The time for elliptic curve point multiplication is 0.0171 s; the time for elliptic curve addition is 0.0061 s; the time for SHA-1 encryption is 0.0005 s; the time for symmetric encryption/decryption is 0.0056 s; and the time for modular exponentiation is 0.057 s. Compared to the calculation time for elliptic curve point multiplication, the calculation time for addition, XOR, and cascade operations is negligible. Table 4 shows the computational time comparison of different protocols in a congested vehicle network. The calculation time comparison of different protocols in non-congested scenarios is shown in Table 5. Figure 5a provides a comparison of the computing overheads of different protocols in congested scenarios, while Figure 5b compares the computing overheads of different protocols in non-congested scenarios.

As shown in Figure 5a, in the congestion scenario, the scheme in this paper has an absolute advantage in the overhead of the tag calculation, which is 66.35% lower than in references [31,32], 66.04% lower than in reference [33], and 65.19% lower than in reference [34]. As shown in Figure 5b, the computational overhead of the scheme in this paper is also relatively small in non-congested scenarios. The overhead of tag calculation is reduced by 32.71% compared to references [31,32], reduced by 32.08% compared to reference [33], and reduced by 30.38% compared to reference [34]. The server computing overhead is reduced by 33.64% compared to reference [31], 33.02% compared to reference [32], 66.19% compared to reference [33], and 52.52% compared to reference [34]. The schemes of reference [31] and reference [32] have very little tag computation overhead and overall computation overhead. Both schemes use only the reader as the intermediary of message exchange without mutual authentication, so although the computational overhead is relatively small, it will lead to other attacks such as man-in-the-middle and simulation attacks. However, the cost of the tag in the scheme in [33] is the same as that of the proposed scheme in terms of general scenarios; however, the total cost of the calculation is relatively large.

### 6.3. Communication Overhead Comparison

**Figure 6:**
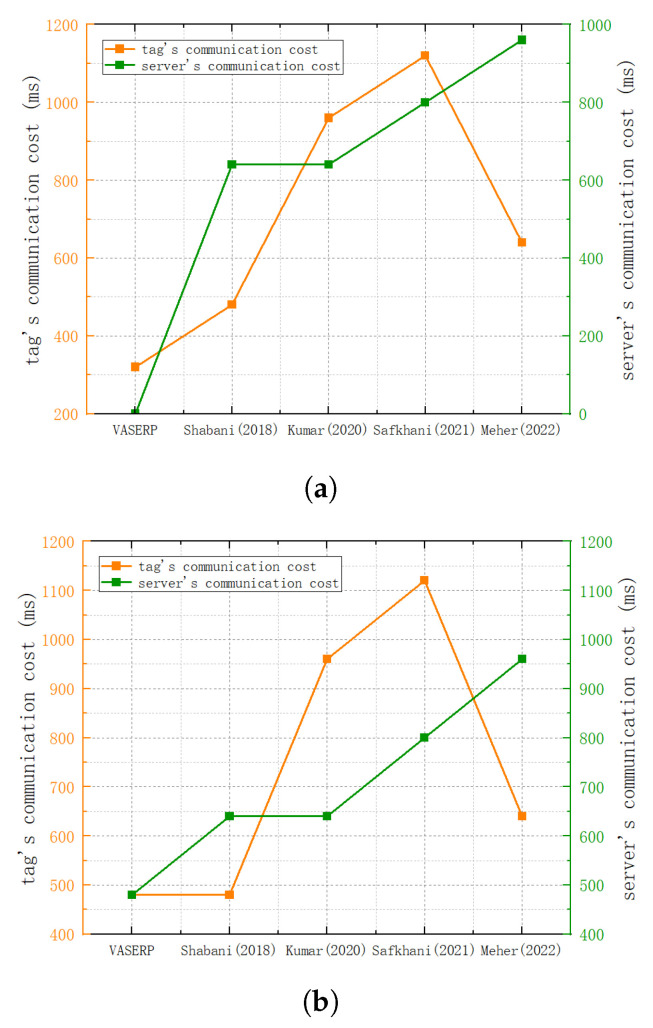
Communication overhead comparison. (**a**) Communication overhead comparison in congestion scenarios; (**b**) comparison of communication overhead in non-congestion scenarios [31,32,33,34].

This section compares the communication overhead of different protocols. Figure 6a shows the specific communication overhead comparison in the congested scenario, and Figure 6b shows the specific communication overhead comparison in the non-congested scenario. In the congested scenario, the communication overhead of our scheme is reduced by 33.3% compared to reference [31], 66.6% compared to reference [32], and 71.42% compared to reference [33]. In the non-congested scenario, the communication overhead of the tag in this scheme is reduced by 50% compared with reference [32] and 57.14% compared with reference [33]. The protocols in both scenarios have significant advantages over other protocols.

### 6.4. Storage Overhead Comparison

**Figure 7:**
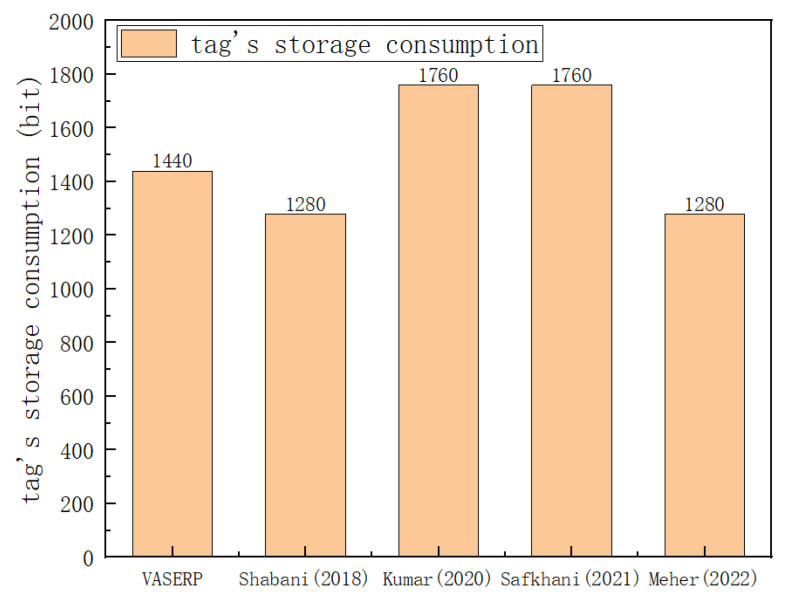
Tag’s storage cost comparison [31,32,33,34].

This section compares the storage overhead of tags and servers. Storage overhead refers to the space required to store parameters on tags, readers, and servers. As the number of tags increases, the storage overhead of the server for the scheme proposed in this work is the same as that of [32,33], while the order of [31] is 1.3 times that of our scheme. Tags need to store $\left\{TID_{i},k,G,P,rid,Q\right\}$. Figure 7 is a comparison of the storage overhead of tags of different protocols. The scheme in this paper has a good advantage in storage.

## 7. Conclusions and Future Work

Aiming at the problems of RFID transmission security, IoV data privacy protection, and fast authentication in traffic congestion scenarios, this article proposes an adaptive, lightweight, and efficient IoV-based authentication scheme. Authentication is performed through the edge server, reducing the cloud’s computing and communication overhead. ECC and hash functions are used to encrypt private data during communication, and this scheme can meet speed and security requirements. Security analysis shows that the scheme in this paper can resist man-in-the-middle attacks, tracking attacks, tag and reader simulation attacks, desynchronization attacks, and other means of attack. Through comparative experiments with other RFID car networking protocols that use the ECC algorithm, the tag calculation overhead in traffic congestion scenarios is reduced by 66.35% compared to other schemes, while the tag calculation overhead in non-congested scenarios is reduced by 32.71%. In terms of communication, the communication overhead of tags and edge servers is also greatly reduced, which improves the authentication efficiency in the IoV. It shows that the scheme proposed in this paper has significant advantages in computing, storage, and communication in congested scenarios. It also achieves a good balance between performance and security in non-congested scenarios.

However, in the IoV, when the RFID reader authenticates passing vehicles, the speed of authenticating a single tag is relatively slow and cannot adapt to the environment of fast-moving vehicles in the IoV. Therefore, subsequent research will be carried out on the reduction of tag computing overhead in the RFID authentication of the IoV and the realization of group authentication of vehicles in the IoV, as well as research on the RFID group authentication protocol for the IoV that uses homomorphic encryption to achieve lower computational overhead.

## Figures and Tables

**Table 1:** Symbols in this scheme.

Symbols	Description
$R_{j}$, $RID_{j}$	the *j*th reader, the identifier of the *j*th reader
$Tag_{i}$, $TID_{i}$	the *i*th tag, the identifier of the *i*th tag
$S_{k}$	*k*th edge server
$T_{i}$	timestamp i=1,2,3……n
$n_{t},n_{r},n_{e}$	the random numbers generated by the tag, reader, and server, respectively
*k*	the key is shared among the edge server, reader, and tag
⊕	XOR operation
||	cascade operation
*p*	the order of a finite field is a large prime number
$F_{p}$	finite field of order *p*
$E_{p}$	elliptic curve over a finite field of large prime order *p*
*G*	the base point of $E_{p}$
*Q*	the public key of the edge server Q=dG
*P*	public key P=kG of reader and tag
*d*	the private key of the edge server, which is a large prime number
*h*	hash function

**Table 2:** Tag index table.

Index	Content
$h(TID_{1})$	$h\left(TID_{1}\right)⊕RID_{j_{1}}$
$h(TID_{2})$	$h\left(TID_{2}\right)⊕RID_{j_{2}}$
…	…
$h(TID_{n})$	$h\left(TID_{n}\right)⊕RID_{j_{n}}$

**Table 3:** Security comparison of different protocols.

Protocol	VASERP	[31]	[32]	[33]	[34]
Tag anonymity	yes	no	yes	yes	no
Resistance against replay attacks	yes	no	no	yes	yes
Resistance against man-in-the-middle attacks	yes	no	no	yes	no
Mutual authentication	yes	yes	yes	yes	no
Forward security	yes	no	yes	yes	yes
Resistance against tag tracking	yes	no	yes	yes	yes
Resistance against impersonation attacks	yes	yes	no	yes	yes
Key agreement protocol	yes	yes	yes	yes	yes
Resistance against desynchronization attacks	yes	yes	yes	yes	yes

**Table 4:** Comparison of computational costs in congested scenarios.

Protocol	Tag’s Computation Cost	Server’s Computation Cost
VASERP	$2T_{h}+T_{ECM}=0.0181s$	0
[31]	$5T_{h}+3T_{ECM}=0.0538s$	$5T_{h}+3T_{ECM}=0.0538s$
[32]	$5T_{h}+3T_{ECM}=0.0538s$	$4T_{h}+3T_{ECM}=0.0533s$
[33]	$4T_{h}+3T_{ECM}=0.0533s$	$6T_{h}+6T_{ECM}=0.1056s$
[34]	$2T_{ECM}+2T_{ECA}+T_{sym}=0.052s$	$3T_{ECM}+3T_{ECA}+T_{sym}=0.0752s$

**Table 5:** Comparison of computational costs in non-congested scenarios.

Protocol	Tag’s Computation Cost	Server’s Computation Cost
VASERP	$4T_{h}+2T_{ECM}=0.0362s$	$3T_{h}+2T_{ECM}=0.0357s$
[31]	$5T_{h}+3T_{ECM}=0.0538s$	$5T_{h}+3T_{ECM}=0.0538s$
[32]	$5T_{h}+3T_{ECM}=0.0538s$	$4T_{h}+3T_{ECM}=0.0533s$
[33]	$4T_{h}+3T_{ECM}=0.0533s$	$6T_{h}+6T_{ECM}=0.1056s$
[34]	$2T_{ECM}+2T_{ECA}+T_{sym}=0.052s$	$3T_{ECM}+3T_{ECA}+T_{sym}=0.0752s$

## Data Availability

Not applicable.

## References

[1] Lai J., Luo C., Wu J., Li J., Wang J., Chen J., Feng G., Song H. (2019). TagSort: Accurate relative localization exploring RFID phase spectrum matching for Internet of Things. IEEE Internet Things J..

[2] Cao B., Gu Y., Lv Z., Yang S., Zhao J., Li Y. (2020). RFID reader anticollision based on distributed parallel particle swarm optimization. IEEE Internet Things J..

[3] Gupta B.B., Gaurav A., Marín E.C., Alhalabi W. (2022). Novel graph-based machine learning technique to secure smart vehicles in intelligent transportation systems. IEEE Trans. Intell. Transp. Syst..

[4] Hammoud A., Otrok H., Mourad A., Dziong Z. (2022). On demand fog federations for horizontal federated learning in IoV. IEEE Trans. Netw. Serv. Manag..

[5] Liang W., Xie S., Cai J., Wang C., Hong Y., Kui X. (2021). Novel private data access control scheme suitable for mobile edge computing. China Commun..

[6] Diao C., Zhang D., Liang W., Li K.C., Hong Y., Gaudiot J.L. (2022). A novel spatial-temporal multi-scale alignment graph neural network security model for vehicles prediction. IEEE Trans. Intell. Transp. Syst..

[7] Song X., Li X., Tang W., Zhang W. (2016). A fusion strategy for reliable vehicle positioning utilizing RFID and in-vehicle sensors. Inf. Fusion.

[8] Qin H., Peng Y., Zhang W. (2017). Vehicles on RFID: Error-cognitive vehicle localization in GPS-less environments. IEEE Trans. Veh. Technol..

[9] Chen R., Huang X., Zhou Y., Hui Y., Cheng N. (2021). UHF-RFID-based real-time vehicle localization in GPS-less environments. IEEE Trans. Intell. Transp. Syst..

[10] Pedraza C., Vega F., Manana G. (2018). PCIV, an RFID-based platform for intelligent vehicle monitoring. IEEE Intell. Transp. Syst. Mag..

[11] Long J., Liang W., Li K.C., Wei Y., Marino M.D. (2022). A Regularized Cross-Layer Ladder Network for Intrusion Detection in Industrial Internet of Things. IEEE Trans. Ind. Inform..

[12] Zhang S., Hu B., Liang W., Li K.C., Gupta B.B. (2023). A Caching-based Dual K-Anonymous Location Privacy-Preserving Scheme for Edge Computing. IEEE Internet Things J..

[13] Liang W., Yang Y., Yang C., Hu Y., Xie S., Li K.C., Cao J. (2022). PDPChain: A consortium blockchain-based privacy protection scheme for personal data. IEEE Trans. Reliab..

[14] Fang W., Li Y., Zhang H., Xiong N., Lai J., Vasilakos A.V. (2014). On the throughput-energy tradeoff for data transmission between cloud and mobile devices. Inf. Sci..

[15] Sandor V.K.A., Lin Y., Li X., Lin F., Zhang S. (2019). Efficient decentralized multi-authority attribute based encryption for mobile cloud data storage. J. Netw. Comput. Appl..

[16] Yang A., Weng J., Yang K., Huang C., Shen X. (2020). Delegating authentication to edge: A decentralized authentication architecture for vehicular networks. IEEE Trans. Intell. Transp. Syst..

[17] Chen M., Chen S., Fang Y. (2017). Lightweight anonymous authentication protocols for RFID systems. IEEE/ACM Trans. Netw..

[18] Fan J., Elmagarmid A.K., Zhu X., Aref W.G., Wu L. (2004). ClassView: Hierarchical video shot classification, indexing, and accessing. IEEE Trans. Multimed..

[19] Aghili S.F., Mala H., Kaliyar P., Conti M. (2019). SecLAP: Secure and lightweight RFID authentication protocol for Medical IoT. Future Gener. Comput. Syst..

[20] Fan K., Luo Q., Zhang K., Yang Y. (2020). Cloud-based lightweight secure RFID mutual authentication protocol in IoT. Inf. Sci..

[21] Fan K., Zhu S., Zhang K., Li H., Yang Y. (2019). A lightweight authentication scheme for cloud-based RFID healthcare systems. IEEE Netw..

[22] Doss R., Trujillo-Rasua R., Piramuthu S. (2020). Secure attribute-based search in RFID-based inventory control systems. Decis. Support Syst..

[23] Lee C.C., Li C.T., Cheng C.L., Lai Y.M., Vasilakos A.V. (2019). A novel group ownership delegate protocol for RFID systems. Inf. Syst. Front..

[24] Tu Y.J., Kapoor G., Piramuthu S. (2022). On Group Ownership Delegate Protocol for RFID Systems. Inf. Syst. Front..

[25] Song J., Harn P.W., Sakai K., Sun M.T., Ku W.S. (2021). An RFID zero-knowledge authentication protocol based on quadratic residues. IEEE Internet Things J..

[26] Rahman F., Hoque M.E., Ahamed S.I. (2017). Anonpri: A secure anonymous private authentication protocol for RFID systems. Inf. Sci..

[27] Liu H., Ning H., Zhang Y., He D., Xiong Q., Yang L.T. (2012). Grouping-proofs-based authentication protocol for distributed RFID systems. IEEE Trans. Parallel Distrib. Syst..

[28] Sun D.Z., Mu Y. (2017). Security of grouping-proof authentication protocol for distributed RFID systems. IEEE Wirel. Commun. Lett..

[29] Yang A., Boshoff D., Hu Q., Hancke G.P., Luo X., Weng J., Mayes K., Markantonakis K. (2021). Privacy-preserving group authentication for rfid tags using bit-collision patterns. IEEE Internet Things J..

[30] Wu F., Li X., Luo X., Gu K. (2022). A novel authentication scheme for edge computing-enabled Internet of Vehicles providing anonymity and identity tracing with drone-assistance. J. Syst. Archit..

[31] Shabani F., Gharaee H., Ghaffari F. An intelligent RFID-enabled authentication protocol in VANET. Proceedings of the 2018 9th International Symposium on Telecommunications (IST).

[32] Kumar V., Ahmad M., Mishra D., Kumari S., Khan M.K. (2020). RSEAP: RFID based secure and efficient authentication protocol for vehicular cloud computing. Veh. Commun..

[33] Safkhani M., Camara C., Peris-Lopez P., Bagheri N. (2021). RSEAP2: An enhanced version of RSEAP, an RFID based authentication protocol for vehicular cloud computing. Veh. Commun..

[34] Meher B.K., Amin R., Das A.K., Khan M.K. (2022). KL-RAP: An Efficient Key-Less RFID Authentication Protocol Based on ECDLP for Consumer Warehouse Management System. IEEE Trans. Netw. Sci. Eng..

[35] Chander B., Gopalakrishnan K. (2022). A secured and lightweight RFID-tag based authentication protocol with privacy-preserving in Telecare medicine information system. Comput. Commun..

[36] Jangirala S., Das A.K., Vasilakos A.V. (2019). Designing secure lightweight blockchain-enabled RFID-based authentication protocol for supply chains in 5G mobile edge computing environment. IEEE Trans. Ind. Inform..

[37] Salem F.M., Amin R. (2020). A privacy-preserving RFID authentication protocol based on El-Gamal cryptosystem for secure TMIS. Inf. Sci..

[38] Shariq M., Singh K., Bajuri M.Y., Pantelous A.A., Ahmadian A., Salimi M. (2021). A secure and reliable RFID authentication protocol using digital schnorr cryptosystem for IoT-enabled healthcare in COVID-19 scenario. Sustain. Cities Soc..

[39] Akram W., Mahmood K., Li X., Sadiq M., Lv Z., Chaudhry S.A. (2022). An energy-efficient and secure identity based RFID authentication scheme for vehicular cloud computing. Comput. Netw..

[40] Chandrakar P., Om H. (2017). A secure and robust anonymous three-factor remote user authentication scheme for multi-server environment using ECC. Comput. Commun..

[41] Lee Y.K., Sakiyama K., Batina L., Verbauwhede I. (2008). Elliptic-curve-based security processor for RFID. IEEE Trans. Comput..

[42] Koblitz N. (1987). Elliptic curve cryptosystems. Math. Comput..

[43] Amin R., Islam S.H., Biswas G., Khan M.K., Kumar N. (2018). A robust and anonymous patient monitoring system using wireless medical sensor networks. Future Gener. Comput. Syst..

